# Anatomical Materiel

**Published:** 1847-02

**Authors:** 


*Anatomical Materiel.*—This last word now stands in the place of *subject for dissection*, and is likely to become familiar to ears medical, through the extra bulletins of the University School in New-York, and the Geneva College in the same great State. This matter was alluded to the week before last, since which, the published documents and a communication from Dr. Webster have been received. Dr. W. is a spirited man, whose energy cannot be put to sleep with mesmeric passes. If he is thoroughly convinced that the University gentlemen have done wrong intentionally, the fur will fly a long while to come. In consequence of having more communications on hand than can possibly be disposed of for a month, we are obliged to forego the publication of Dr. Webster's paper on the *materiel* case for the present week.—*Ibid.*

